# Cure and Curse: *E. coli* Heat-Stable Enterotoxin and Its Receptor Guanylyl Cyclase C

**DOI:** 10.3390/toxins2092213

**Published:** 2010-08-26

**Authors:** Philipp R. Weiglmeier, Paul Rösch, Hanna Berkner

**Affiliations:** Lehrstuhl für Biopolymere und Forschungszentrum für Bio-Makromoleküle, Universität Bayreuth, Universitätsstraße 30, 95447 Bayreuth, Germany; Email: philipp.weiglmeier@uni-bayreuth.de (P.R.W.); roesch@unibt.de (P.R.)

**Keywords:** heat-stable enterotoxin, guanylyl cyclase C, secretory diarrhea, colorectal cancer

## Abstract

Enterotoxigenic *Escherichia coli* (ETEC) associated diarrhea is responsible for roughly half a million deaths per year, the majority taking place in developing countries. The main agent responsible for these diseases is the bacterial heat-stable enterotoxin STa. STa is secreted by ETEC and after secretion binds to the intestinal receptor guanylyl cyclase C (GC-C), thus triggering a signaling cascade that eventually leads to the release of electrolytes and water in the intestine. Additionally, GC-C is a specific marker for colorectal carcinoma and STa is suggested to have an inhibitory effect on intestinal carcinogenesis. To understand the conformational events involved in ligand binding to GC-C and to devise therapeutic strategies to treat both diarrheal diseases and colorectal cancer, it is paramount to obtain structural information on the receptor ligand system. Here we summarize the currently available structural data and report on physiological consequences of STa binding to GC-C in intestinal epithelia and colorectal carcinoma cells.

## 1. Introduction

Enterotoxigenic *Escherichia coli* (ETEC) are a major cause for acute secretory diarrhea in developing countries with insufficient sanitation and no adequate supply of clean water. These pathogens account for around a half million deaths per year, mostly of children in developing countries [1], and are the cause of traveller’s diarrhea. ETEC, a very diverse group of pathogenic *E. coli*, colonize the small intestine and produce the toxic agents heat-labile (LT) and heat-stable (ST) enterotoxin. Apparently, the acquisition of enterotoxin genes is sufficient for the expression of enterotoxigenicity [2]. Clinically, ETEC associated diarrhea is virtually indistinguishable from cholera [3]. The *E. coli* heat-labile toxin LT is a hexameric protein consisting of a single A subunit and a homopentameric B subunit. The two domain A subunit represents the actual toxin, which activates the guanine nucleotide protein Gsα by ADP-ribosylation and ultimately leads to stimulated secretion by a cAMP-dependent mechanism involving the cystic fibrosis transmembrane conductance regulator (CFTR). LT is structurally and functionally very similar to cholera toxin (CT) [4]. Heat-stable enterotoxins are small peptides that are secreted by enterotoxigenic bacteria. ST peptides are active even after 60 min of heating at 95 °C [5]. Two classes of STs that differ in structure and function can be distinguished: the methanol soluble protease resistant and guanylyl cyclase C (GC-C) binding STa and the methanol insoluble and protease sensitive STb. STb is a 48 amino acid peptide associated with disease in cattle, but not in humans, and it does not bind to GC-C. STb was shown to increase intracellular levels of Ca^2+^ [6].

STas are 18 or 19 amino acid cysteine-rich peptides that activate intestinal guanylyl cyclase C and induce secretion by cGMP-dependent activation of CFTR. Genetically, the toxins are encoded on plasmids [7]. In some cases they were found to be encoded in transposons or in sequences that were once part of a transposon [8,9].

Peptides that are highly homologous to *E. coli* STa have been identified in other enteric bacteria such as *Klebsiella pneumoniae* [10], *Yersinia enterolitica* [11], *Citrobacter freundii* [12], cholera toxin positive *Vibrio cholerae* O1 [13] and nonagglutinable *Vibrios* [14,15,16].

Other notable enterotoxins produced by pathogenic *E. coli* include the enteroaggregative heat-stable toxin EAST1 [17,18] which is structurally similar to STa, and Shiga toxin Stx produced by shiga-toxigenic *E. coli* (STEC) [19].

## 2. Structure of ST Peptides

The two most common STas produced by *E. coli* are STh and STp. These toxins consist of 18 (STp) or 19 (STh) amino acids including six cysteines that form three intramolecular disulfide linkages. Both peptides share the carboxy-terminal 14 residues which are sufficient for enterotoxicity and referred to as the toxic domain [20]. This toxic domain shows significant homology to the sequence of the mammalian endogenous peptides guanylin [21], uroguanylin [22] and lymphoguanylin [23]. In contrast, the amino-terminal four or five residues are neither homologous in the two ST species nor required for biological activity.

**Figure 1:**
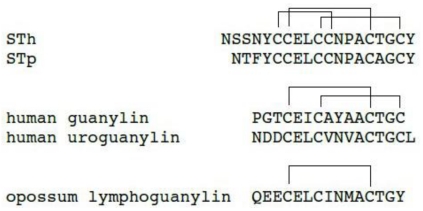
The primary structure of *E. coli* STh and STp, human hormones guanylin, uroguanylin and opossum lymphoguanylin. Disulfide linkages are indicated by lines above.

The mode of disulfide bond formation is identical in both STh and STp (Figure 1): In the STh numbering system, the bonds are Cys6–Cys11, Cys7–Cys15 and Cys10–Cys18 [24]. In order to determine the relative importance of each disulfide bond, STh analogs were synthesized that lack one or two disulfide linkages by pairwise replacement of cysteines with alanines [25]. Comparison of the ability of these analogs to inhibit the binding of radiolabeled [^125^I-Y4]-STh(6–18) to intestinal cells revealed that the Cys7–Cys15 bond is crucial for biological activity. Replacement of the Cys6–Cys11 and Cys10–Cys18 bond results in peptides that bind 4200 and 130 times less strongly to their receptor, respectively, as measured by their IC_50_ values. The Cys7–Cys15 disulfide linkage alone, however, is not sufficient for binding [25,26].

The homologous mammalian peptides guanylin and uroguanylin both contain four cysteine residues that form two disulfide bonds with 1–3/2–4 connectivity (Figure 1), lacking one of the bonds present in *E. coli* STa. The two cysteine bonds give rise to topological isomerism in both peptides [27,28]. Only one of the interconvertible topoisomers of each peptide, the so-called A-form, is biologically active and binds to GC-C. The rate of interconversion differs in guanylin and uroguanylin. Guanylin interconversion occurs with a half-life of seconds while the process is much slower for uroguanylin [29]. The interconversion was shown to be controlled by sterical hindrance from the carboxy-terminal residues as well as by the side-chains in the central part of the peptides that modulates the flexibility of the chains [30,31]. 

Both STh and STp are synthesized as 72 amino acid precursor proteins consisting of a pre signal peptide, a pro sequence, and the carboxy-terminal enterotoxin [32,33]. The polypeptide is synthesized as an intracellular pre-pro-STa. The 19 amino acid signal sequence is cleaved during translocation from the cytoplasm to the periplasm by a SecA-dependent export pathway [34]. The exact function of the pro sequence, which is non-essential for enterotoxin secretion, is unclear [32], although the highly conserved region from residue 29 to 38 in the pro sequence greatly increases the translocation of STa across the inner membrane [35]. If amino acid residues 29 to 31 (K-E-K) are substituted by hydrophobic residues or by all-basic residues (K-K-K), the efficiency of STa delivery to the periplasm is significantly reduced. Conversely, an amino acid substitution to an all-acidic motif (E-E-E) leads to increased translocation of STa across the inner membrane. Some studies indicated that the pro sequence is cleaved off inside the periplasm where the thiol-disulfide oxidoreductase DsbA is thought to catalyse the disulfide bond formation in STa [36,37]. The mature STa is then believed to be secreted across the outer membrane. Other results are in conflict with this view as deletion of the pro sequence or the STa peptide leads to detection of STa or pro peptide in the supernatant of cells, clearly indicating that the pro region can cross the outer membrane [38]. Other studies showed that various fusion proteins consisting of STa and the major subunit ClpG of *E. coli* CS31A fimbriae antigen are secreted across the outer membrane and that the disulfide bonds in the bioactive STa were formed extracellularly by a DsbA-independent mechanism possibly involving molecular oxygen [39,40]. However, these authors also showed that the export of their fusion proteins was dependent on the CS31A secretion pathway and might differ substantially from the secretion of native STa.

The molecular structure of the toxic domain of STa has been studied by NMR [41] and X-ray crystallography [42,43,44]. The STp molecule is arranged as a right-handed spiral assembled by three structural elements: a 3_10_-helix from Cys5 to Cys9, a type I β-turn from Asn11 to Cys14, and a type II β-turn from Cys14 to Cys17 [42]. This structure is stabilized by the three disulfide linkages as well as by hydrogen bonds formed by the NH groups of most of the constituent amino acids. The latter include inter- as well as intra-turn 4–1 type bonds. The Cys5–Cys10 disulfide linkage adopts a rare right-handed conformation while the other two disulfide linkages have a left-handed spiral conformation [43] which is most common in proteins [45]. All the amino acid side chains are oriented to the outside, giving the molecule a hydrophobic character. The completely exposed β-turn formed by amino acids Asn11, Pro12, and Ala13 [41] is thought to constitute the receptor binding site of STa as point mutations in this region substituting Asn11 by Ala or Ala13 by D-Ala dramatically reduced the receptor binding activity of STa [46]. On the other hand, a Pro12Gly substitution, which is thought to disturb the turn conformation, has only a limited effect on binding activity [47]. In addition, comparative molecular field analysis predicted that the amide backbone of Cys5-Cys6-Glu7-Leu8 plays an important role in the interaction with GC-C [48]. In a recent study, a homohexameric ring-shaped structure of STp(5–17) was observed [44]. In this structure, the interface between adjacent monomers is formed by the 3_10_-helix of one peptide and the type II β-turn of its neighbor whereas the putative binding site Asn11-Pro12-Ala13 is oriented towards the exterior. Hydrophilic groups are concealed. This gave rise to the suggestion that the hexamer is the native structure recognized by GC-C, however, the hexamer formation may be a result of the hydrophobic solvent chosen in this particular crystallographic study.

**Figure 2:**
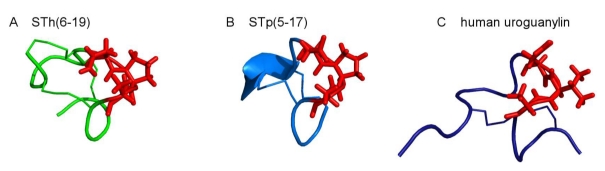
Comparison of (**A**) the solution structure of STh(6–19)[41]; (**B**) the crystal structure of STp(5–17)[42], PDB: 1ETN; and (**C**) the solution structure of human uroguanylin [28], PDB: 1UYA. The hydrophobic region implicated in receptor binding is depicted as red sticks in each structure.

Overall, the crystal structure of STp(5–17) [42] and the solution structure of STh(6–19) determined by NMR [41] are in good agreement despite a different orientation of the carboxy termini (Figure 2). Signal broadening of resonances of the amino-terminal residues indicates conformational dynamics in the µs-ms timescale [41,49].

The structure of STa is very similar to the structure of the biologically active A-forms of guanylin and uroguanylin. The additional disulfide bond in STa prevents a similar topological isomerism.

## 3. The Heat-Stable Enterotoxin Receptor: Intestinal GC-C

The receptor for STa was shown to be intestinal GC-C [50,51]. This 1050 amino acid membrane protein is almost exclusively expressed on the brush border membrane of epithelial cells in the small intestine and the colon [52]. GC-C expression is uniformly distributed along the crypt-villus axis in the small intestine. However, in the colon, GC-C is mainly restricted to the crypts [53]. The transcription factors CDX2, a caudal family homeodomain protein generally involved in differentiation processes, and hepatocyte nuclear factor-4 (HNF-4), a zinc-finger-containing nuclear hormone receptor, regulate expression of GC-C [54,55]. Besides its function in electrolyte homeostasis, GC-C signaling coordinates proliferation, migration, differentiation and apoptosis in the crypt-villus axis [56].

The primary structure of GC-C [57] indicates that the protein consists of an amino-terminal 23 residue signal peptide, followed by an extracellular domain (ECD), a single transmembrane helix, a kinase homology domain (KHD), a linker region, the actual catalytic guanylyl cyclase domain, and a carboxy-terminal domain [58]. The signal peptide is proteolytically removed during maturation. Other members of the so-called mammalian particulate guanylyl cyclase family include the natriuretic peptide receptors GC-A and GC-B, sensory guanylyl cyclases GC-D, GC-E and GC-F as well as the less studied murine renal GC-G [59]. The latter four GCs have no known ligands and are therefore termed orphan receptors. Whereas the intracellular domains of particulate GCs are closely homologous, the similarity of the extracellular domains is not very pronounced, and, e.g., for GC-C and GC-A the sequence identity is only 19%. A homology model of the ECD of GC-C, based on the known structure of GC-A [60] and experimental data on the configuration of the disulfide linkages, suggests that the ECD consists of two subdomains: one membrane-distal and mainly α-helical and the other one membrane-proximal and predominantly β-sheet type [61]. Indeed, the membrane proximal domain expressed as a single fragment can fold independently [62] and seems to contain the STa binding site [63,64]. The intracellular kinase homology domain plays an important regulatory role. Phylogenetic studies revealed an apparent co-evolution of the KHD and the catalytic domain that also includes the linker region between both domains [65]. The KHD is thought to provide an inhibitory effect on the catalytic domain, which is only relieved by binding of a ligand to the ECD. Consequently, deletion of the KHD results in a constitutive ligand-independent activation of the GC domain [66]. The KHD does bind ATP although it is assumed to be catalytically inactive for lack of the highly conserved H-R-D motif essential for protein kinase activity [67]. The catalytic domain is highly homologous in all particulate as well as the soluble guanylyl cyclases, and it is very similar to the catalytic domain of adenylyl cyclases.

GC-C is a heavily glycosylated protein [68,69] that contains 8–10 *N*-linked glycosylation sites, depending on the species. It is not clear whether the sugar residues are essential for ligand binding. Two differently glycosylated forms of GC-C of 130 kDa and 145 kDa bind STa with the same affinity, although only the mature fully glycosylated 145 kDa GC-C is activated by ligand binding [70]. Since enzymatic deglycosylation of mature GC-C has no effect on binding affinity and activation, the sugar residues might be needed for correct protein folding rather than be directly involved in ligand binding. Moreover, mutagenic removal of specific *N*-glycosylation sites from the extracellular domain of an insect-baculovirus system expressed GC-C [71] resulted in a drop in binding capacity but not in binding affinity, supporting the notion that without certain sugar residues GC-C does not adopt a stable conformation that allows for ligand recognition [72]. However, in the same study, deglycosylation of GC-C by PNGase F resulted in a loss of STa binding.

As in adenylyl cyclases, the catalytic domain of GC-C is only active as a dimer. However, whereas adenylyl cylcases and soluble guanylyl cyclases are heterodimers, the active center of particulate guanylyl cyclase is homodimeric with the linker region between KHD and GC domain providing the necessary dimerization motif. GC-C might actually be present as a homotrimer [73,74] with only two subunits forming the catalytic center on activation. Since the extracellular domains of both GC-A [60] and the homologous atrial natriuretic peptide clearance receptor NPR-C [75] are dimeric, it is possible that the ECD of GC-C also forms oligomers.

## 4. Regulation of Intestinal Fluid Secretion and STa-Induced Diarrhea

**Figure 3:**
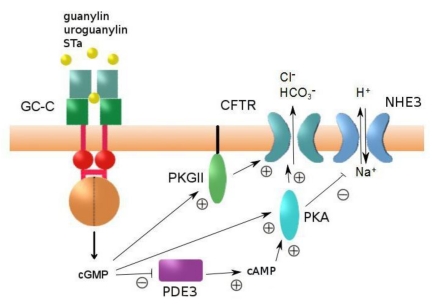
Schematic representation of intestinal secretion regulated by GC-C. PDE3: cGMP-inhibitable phosphodiesterase 3; PKGII: cGMP-dependent protein kinase II; PKA: cAMP-dependent protein kinase; CFTR: cystic fibrosis transmembrane conductance regulator; NHE: Na/H exchanger.

GC-C is a key receptor in regulating the electrolyte level and the fluidity of the intestinal content. Binding of either an endogenous or exogenous ligand to the ECD of GC-C triggers a conformational event, which leads to activation of the catalytic domain and the formation of cGMP. This initiates a signaling cascade that ultimately results in secretion of electrolytes into the intestinal lumen accompanied by water release (Figure 3). Overactivation of GC-C by STa is the physiological basis of ETEC induced watery diarrhea.

The main mediator of chloride secretion is the cystic fibrosis transmembrane conductance regulator (CFTR), a chloride channel present at the apical membrane of intestinal brush-border epithelial cells [76]. Increased levels of cGMP activate the cGMP-dependent protein kinase II (PKGII), which co-localizes with the CFTR and phosphorylates it [77,78], thus promoting the electrogenic release of Cl^−^ into the lumen [76]. In addition, cGMP is able to inhibit phosphodiesterase 3 (PDE3). PDE3 hydrolyzes cAMP, and its inhibition results in accumulation of cAMP which, in turn, activates protein kinase A, providing an additional PKGII-independent mechanism to stimulate Cl^−^ secretion [79]. cAMP as well as cGMP can trigger an increased targeting of CFTR from intracellular vesicles to the cell membrane [80,81,82].

The Na^+^/H^+^-exchanger (NHE) is a second target of cGMP action in intestinal epithelia. PKA inhibits the re-absorption of sodium by NHE [83].

Although this mechanism is well established, it was argued recently that water accumulation in the intestinal lumen associated with STa is not as much a result of an active secretion of electrolytes as it is a consequence of impaired fluid absorption [84,85]. It was stressed that the available evidence does not unambiguously point to an increased electrolyte secretion following STa administration. 

As main regulator of intestinal fluidity, GC-C is a therapeutic target for treatment of constipation related disorders. Linaclotide, an agonist of GC-C, is closely related to STa and used as an investigational drug against constipation-predominant irritable bowel syndrome (IBS-C) and chronic constipation [86,87].

GC-C and its ligands are also implicated in regulation of the intestinal pH, since CFTR does not only transport Cl^−^ but also HCO_3_^−^. Regulation of the pH in the intestine is crucial because a low pH can result in tissue damage. Surprisingly, it was shown that STa can induce HCO_3_^−^ secretion independently of PKGII and CFTR. In CF mice that lack functional CFTR, this secretion is electroneutral and dependent on Cl^−^, which suggests involvement of a Cl^−^/HCO_3_^−^ exchanger [88]. In GC-C knockout mice an alternative STa receptor is present that causes bicarbonate secretion by a downstream signal cascade different from the PKGII and CFTR mechanism [89].

The possibility of an alternative receptor for STa and guanylin peptides has been investigated very early [90,91]. GC-C knockout mice are not only healthy, fertile and have a normal responsiveness to other secretory signals, they also show statistically significant, albeit strongly reduced, STa binding [92,93]. However, in these animals, no secretion is induced by STa. A non-GC-C, G protein-coupled receptor for guanylin, uroguanylin and STa has been reported in the kidney [94,95].

## 5. Involvement of GC-C in Colorectal Cancer

Expression of the endogenous GC-C ligands guanylin and uroguanylin is lost in transformed human colon cancer cells [96]. In contrast, GC-C can be found in all types of primary and metastatic colorectal cancer cells [97,98]. Thus, GC-C is a very specific marker for colorectal carcinoma cells that can be exploited for PCR-based tumor detection strategies [99,100]. Due to their very restricted expression outside of intestinal epithelia and their ubiquitous presence on colorectal carcinoma GC-C derived antigens promise to be an important target for immunotherapy of metastatic cancer [101].

Furthermore, its ability to bind GC-C with very high affinity makes STa a useful template structure for synthesis of peptide analogs that target and visualize cancer, for instance by CT imaging [102,103,104,105,106,107]. In addition, the potential use of structurally less demanding uroguanylin analogs has been investigated [108].

Since STa is internalized upon binding of GC-C in T84 cells, it might be used to deliver diagnostic or therapeutic drugs into colorectal carcinoma cells [109].

Recently, it has been hypothesized that STa is not only suitable for efficient targeting of colorectal carcinoma cells but may also prevent proliferation of these cells and thus inhibit initiation and progression of colon cancer [110]. Indeed, an inverse relationship between the global incidence of colorectal cancer and the ETEC infection frequency can be found. While colorectal cancer is a common cause of mortality in Western societies it is rather rare in developing countries [111]. This striking observation together with the apparent ability of STa and uroguanylin to delay the progression of the cell cycle in human colon cancer cells via a cGMP- and Ca^2+^-dependent pathway [110,112,113] lead to a new view on the events involved in colorectal tumorigenesis. It was speculated that lack or insufficient expression of the paracrine hormones guanylin and uroguanylin affecting GC-C and its downstream effectors are at the very beginning of cancer formation in intestinal cells [114]. As mentioned above, the guanylin peptides are frequently lost in very early stages of colorectal carcinogenesis. Inversely, their receptor GC-C is even overexpressed in colorectal tumors, probably in order to compensate hormone deficiency [115]. GC-C’s transcriptional regulator CDX2 is overexpressed in certain forms of colorectal cancer as well [116]. At first, this discovery seemed to question the role of both proteins as tumor suppressors, but *in vivo* experiments with mice showed that elimination of the two proteins promotes both tumor initiation and growth [117]. Furthermore, differential microarray analysis revealed that interruption of GC-C signaling leads to activation of pro-oncogenic signaling by the AKT pathway [118]. Thus, the mechanism underlying GC-C’s function as a tumor suppressor seems to be the control of proliferation and metabolism of the intestinal epithelium in order to maintain genetic stability [119]. As disruption of GC-C signaling in the earliest stages of colorectal cancer is a consequence of hormone insufficiency, it is to be assumed that hormone replacement therapy may be able to prevent or treat colon cancer. Actually, feeding of uroguanylin to Apc^Min/+^ mice, an animal model for colon cancer, leads to a reduction of both tumor number and size [120]. Being an agonist of uroguanylin and guanylin, STa also results in inhibition of proliferation in human colorectal cancer cells [110,112,121].

## 6. Outlook

The previous paragraphs underline that recent research has helped to transform STa’s reputation from that of a curse torturing or even killing thousands of people in developing countries, to that of a cure for less severe as well as life-threatening diseases. STa analogs for treatment of constipation are already in the late phase of clinical trials and uroguanylin as well as different STa analogs that target colon cancer therapy might soon follow.

However, a reliable cure for acute secretory diarrhea elicited by STa is still missing. One reason for this might be the lack of structural and mechanistic information as to STa receptor interaction, and such data on GC-C and the GC-C:STa complex would be extremely valuable. Consequently, an important step towards the development of a remedy against STa mediated diarrhea would be the determination of the three-dimensional structure of the ligand-binding extracellular domain of GC-C. Comparison of the structures in absence and in presence of STa could help to elucidate the activation mechanism. These data could pave the way to the design of either antagonistic STa analogs blocking the receptor or scavengers competing with it for STa binding.

## References

[1] World Health Organization (2006). Future Directions for Research on Enterotoxigenic *Escherichia coli* Vaccines for Developing Countries. Wkly. Epidemiol. Rec..

[2] Turner S.M., Chaudhuri R.R., Jiang Z.D., DuPont H., Gyles C., Penn C.W., Pallen M.J., Henderson I.R. (2006). Phylogenetic Comparisons Reveal Multiple Acquisitions of the Toxin Genes by Enterotoxigenic *Escherichia coli* Strains of Different Evolutionary Lineages. J. Clin. Microbiol..

[3] Sack D.A., McLaughlin J.C., Sack R.B., Orskov F., Orskov I. (1977). Enterotoxigenic *Escherichia coli* Isolated from Patients at a Hospital in Dacca. J. Infect. Dis..

[4] Spangler B.D. (1992). Structure and Function of Cholera Toxin and the Related *Escherichia coli* Heat-Labile Enterotoxin. Microbiol. Rev..

[5] Sack R.B. (1975). Human Diarrheal Disease Caused by Enterotoxigenic *Escherichia coli*. Annu. Rev. Microbiol..

[6] Dreyfus L.A., Harville B., Howard D.E., Shaban R., Beatty D.M., Morris S.J. (1993). Calcium Influx Mediated by the *Escherichia coli* Heat-Stable Enterotoxin B (STB). Proc. Natl. Acad. Sci. USA.

[7] Moseley S.L., Samadpour-Motalebi M., Falkow S. (1983). Plasmid Association and Nucleotide Sequence Relationships of Two Genes Encoding Heat-Stable Enterotoxin Production in *Escherichia coli* H-10407. J. Bacteriol..

[8] So M., McCarthy B.J. (1980). Nucleotide Sequence of the Bacterial Transposon Tn1681 Encoding a Heat-Stable (ST) Toxin and Its Identification in Enterotoxigenic *Escherichia coli* Strains. Proc. Natl. Acad. Sci. USA.

[9] Dallas W.S. (1990). The Heat-Stable Toxin I Gene from *Escherichia coli* 18D. J. Bacteriol..

[10] Klipstein F.A., Engert R.F., Houghten R.A. (1983). Immunological Properties of Purified *Klebsiella pneumoniae* Heat-Stable Enterotoxin. Infect. Immun..

[11] Takao T., Tominaga N., Shimonishi Y., Hara S., Inoue T., Miyama A. (1984). Primary Structure of Heat-Stable Enterotoxin Produced by Yersinia Enterocolitica. Biochem. Biophys. Res. Commun..

[12] Guarino A., Capano G., Malamisura B., Alessio M., Guandalini S., Rubino A. (1987). Production of *Escherichia coli* STa-Like Heat-Stable Enterotoxin by *Citrobacter freundii* Isolated from Humans. J. Clin. Microbiol..

[13] Takeda T., Peina Y., Ogawa A., Dohi S., Abe H., Nair G.B., Pal S.C. (1991). Detection of Heat-Stable Enterotoxin in a Cholera Toxin Gene-Positive Strain of *Vibrio cholerae* O1. FEMS Microbiol. Lett..

[14] Takao T., Shimonishi Y., Kobayashi M., Nishimura O., Arita M., Takeda T., Honda T., Miwatani T. (1985). Amino Acid Sequence of Heat-Stable Enterotoxin Produced by *Vibrio cholerae* Non-01. FEBS Lett..

[15] Arita M., Takeda T., Honda T., Miwatani T. (1986). Purification and Characterization of *Vibrio cholerae* Non-O1 Heat-Stable Enterotoxin. Infect. Immun..

[16] Huang X., Yoshino K., Nakao H., Takeda T. (1997). Nucleotide Sequence of a Gene Encoding the Novel Yersinia Enterocolitica Heat-Stable Enterotoxin that Includes a Pro-Region-Like Sequence in Its Mature Toxin Molecule. Microb. Pathog..

[17] Savarino S.J., Fasano A., Robertson D.C., Levine M.M. (1991). Enteroaggregative *Escherichia coli* Elaborate a Heat-Stable Enterotoxin Demonstrable in an *in Vitro* Rabbit Intestinal Model. J. Clin. Invest..

[18] Savarino S.J., Fasano A., Watson J., Martin B.M., Levine M.M., Guandalini S., Guerry P. (1993). Enteroaggregative *Escherichia coli* Heat-Stable Enterotoxin 1 Represents another Subfamily of *E. coli* Heat-Stable Toxin. Proc. Natl. Acad. Sci. USA.

[19] Paton J.C., Paton A.W. (1998). Pathogenesis and Diagnosis of Shiga Toxin-Producing *Escherichia coli* Infections. Clin. Microbiol. Rev..

[20] Yoshimura S., Ikemura H., Watanabe H., Aimoto S., Shimonishi Y., Hara S., Takeda T., Miwatani T., Takeda Y. (1985). Essential Structure for Full Enterotoxigenic Activity of Heat-Stable Enterotoxin Produced by Enterotoxigenic *Escherichia coli*. FEBS Lett..

[21] Currie M.G., Fok K.F., Kato J., Moore R.J., Hamra F.K., Duffin K.L., Smith C.E. (1992). Guanylin: An Endogenous Activator of Intestinal Guanylate Cyclase. Proc. Natl. Acad. Sci. USA.

[22] Hamra F.K., Forte L.R., Eber S.L., Pidhorodeckyj N.V., Krause W.J., Freeman R.H., Chin D.T., Tompkins J.A., Fok K.F., Smith C.E. (1993). Uroguanylin: Structure and Activity of a Second Endogenous Peptide that Stimulates Intestinal Guanylate Cyclase. Proc. Natl. Acad. Sci. USA.

[23] Forte L.R., Eber S.L., Fan X., London R.M., Wang Y., Rowland L.M., Chin D.T., Freeman R.H., Krause W.J. (1999). Lymphoguanylin: Cloning and Characterization of a Unique Member of the Guanylin Peptide Family. Endocrinology.

[24] Shimonishi Y., Hidaka Y., Koizumi M., Hane M., Aimoto S., Takeda T., Miwatani T., Takeda Y. (1987). Mode of Disulfide Bond Formation of a Heat-Stable Enterotoxin (STh) Produced by a Human Strain of Enterotoxigenic *Escherichia coli*. FEBS Lett..

[25] Gariepy J., Judd A.K., Schoolnik G.K. (1987). Importance of Disulfide Bridges in the Structure and Activity of *Escherichia coli* Enterotoxin ST1b. Proc. Natl. Acad. Sci. USA.

[26] Yamasaki S., Hidaka Y., Hideaki I., Takeda Y., Shimonishi Y. (1988). Structural Requirements for the Spatial Structure and Toxicity of Heat-Stable Enterotoxin (ST_h_) of Enterotoxigenic *Escherichia Coli*. Bull. Chem. Soc. Jpn..

[27] Skelton N.J., Garcia K.C., Goeddel D.V., Quan C., Burnier J.P. (1994). Determination of the Solution Structure of the Peptide Hormone Guanylin: Observation of a Novel Form of Topological Stereoisomerism. Biochemistry.

[28] Marx U.C., Klodt J., Meyer M., Gerlach H., Rosch P., Forssmann W.G., Adermann K. (1998). One Peptide, Two Topologies: Structure and Interconversion Dynamics of Human Uroguanylin Isomers. J. Pept. Res..

[29] Klodt J., Kuhn M., Marx U.C., Martin S., Rosch P., Forssmann W.G., Adermann K. (1997). Synthesis, Biological Activity and Isomerism of Guanylate Cyclase C-Activating Peptides Guanylin and Uroguanylin. J. Pept. Res..

[30] Schulz A., Escher S., Marx U.C., Meyer M., Rosch P., Forssmann W.G., Adermann K. (1998). Carboxy-Terminal Extension Stabilizes the Topological Stereoisomers of Guanylin. J. Pept. Res..

[31] Schulz A., Marx U.C., Tidten N., Lauber T., Hidaka Y., Adermann K. (2005). Side Chain Contributions to the Interconversion of the Topological Isomers of Guanylin-Like Peptides. J. Pept. Sci..

[32] Okamoto K., Takahara M. (1990). Synthesis of *Escherichia coli* Heat-Stable Enterotoxin STp as a Pre-Pro form and Role of the Pro Sequence in Secretion. J. Bacteriol..

[33] Rasheed J.K., Guzman-Verduzco L.M., Kupersztoch Y.M. (1990). Two Precursors of the Heat-Stable Enterotoxin of *Escherichia coli*: Evidence of Extracellular Processing. Mol. Microbiol..

[34] Pugsley A.P. (1993). The Complete General Secretory Pathway in Gram-Negative Bacteria. Microbiol. Rev..

[35] Yamanaka H., Fuke Y., Hitotsubashi S., Fujii Y., Okamoto K. (1993). Functional Properties of Pro Region of *Escherichia coli* Heat-Stable Enterotoxin. Microbiol. Immunol..

[36] Yamanaka H., Kameyama M., Baba T., Fujii Y., Okamoto K. (1994). Maturation Pathway of *Escherichia coli* Heat-Stable Enterotoxin I: Requirement of DsbA for Disulfide Bond Formation. J. Bacteriol..

[37] Yamanaka H., Nomura T., Fujii Y., Okamoto K. (1997). Extracellular Secretion of *Escherichia coli* Heat-Stable Enterotoxin I across the Outer Membrane. J. Bacteriol..

[38] Yang Y., Gao Z., Guzman-Verduzco L.M., Tachias K., Kupersztoch Y.M. (1992). Secretion of the STA3 Heat-Stable Enterotoxin of *Escherichia coli*: Extracellular Delivery of Pro-STA Is Accomplished by either Pro or STA. Mol. Microbiol..

[39] Batisson I., der Vartanian M. (2000). Extracellular DsbA-Insensitive Folding of *Escherichia coli* Heat-Stable Enterotoxin STa *in Vitro*. J. Biol. Chem..

[40] Batisson I., der Vartanian M., Gaillard-Martinie B., Contrepois M. (2000). Full Capacity of Recombinant *Escherichia coli* Heat-Stable Enterotoxin Fusion Proteins for Extracellular Secretion, Antigenicity, Disulfide Bond Formation, and Activity. Infect. Immun..

[41] Matecko I., Burmann B.M., Schweimer K., Kalbacher H., Einsiedel J., Gmeiner P., Rösch P. (2008). Structural Characterization of the *E. Coli* Heat Stable Enterotoxin STh. Open Spectrosc. J..

[42] Ozaki H., Sato T., Kubota H., Hata Y., Katsube Y., Shimonishi Y. (1991). Molecular Structure of the Toxin Domain of Heat-Stable Enterotoxin Produced by a Pathogenic Strain of *Escherichia coli*. A Putative Binding Site for a Binding Protein on Rat Intestinal Epithelial Cell Membranes. J. Biol. Chem..

[43] Sato T., Ozaki H., Hata Y., Kitagawa Y., Katsube Y., Shimonishi Y. (1994). Structural Characteristics for Biological Activity of Heat-Stable Enterotoxin Produced by Enterotoxigenic *Escherichia coli*: X-ray Crystallography of Weakly Toxic and Nontoxic Analogs. Biochemistry.

[44] Sato T., Shimonishi Y. (2004). Structural Features of *Escherichia coli* Heat-Stable Enterotoxin that Activates Membrane-Associated Guanylyl Cyclase. J. Pept. Res..

[45] Richardson J.S. (1981). The Anatomy and Taxonomy of Protein Structure. Adv. Protein Chem..

[46] Carpick B.W., Gariepy J. (1991). Structural Characterization of Functionally Important Regions of the *Escherichia coli* Heat-Stable Enterotoxin STIb. Biochemistry.

[47] Waldman S.A., O'Hanley P. (1989). Influence of a Glycine or Proline Substitution on the Functional Properties of a 14-Amino-Acid Analog of *Escherichia coli* Heat-Stable Enterotoxin. Infect. Immun..

[48] Wolfe H.R., Waldman S.A. (2002). A Comparative Molecular Field Analysis (COMFA) of the Structural Determinants of Heat-Stable Enterotoxins Mediating Activation of Guanylyl Cyclase C. J. Med. Chem..

[49] Gariepy J., Lane A., Frayman F., Wilbur D., Robien W., Schoolnik G.K., Jardetzky O. (1986). Structure of the Toxic Domain of the *Escherichia coli* Heat-Stable Enterotoxin ST I. Biochemistry.

[50] Schulz S., Green C.K., Yuen P.S., Garbers D.L. (1990). Guanylyl Cyclase Is a Heat-Stable Enterotoxin Receptor. Cell.

[51] Vaandrager A.B., Schulz S., de Jonge H.R., Garbers D.L. (1993). Guanylyl Cyclase C Is an *N*-Linked Glycoprotein Receptor that Accounts for Multiple Heat-Stable Enterotoxin-Binding Proteins in the Intestine. J. Biol. Chem..

[52] de Jonge H.R. (1975). Properties of Guanylate Cyclase and Levels of Cyclic GMP in Rat Small Intestinal Villous and Crypt Cells. FEBS Lett..

[53] Basu N., Arshad N., Visweswariah S.S. (2010). Receptor Guanylyl Cyclase C (GC-C): Regulation and Signal Transduction. Mol. Cell. Biochem..

[54] Di Guglielmo M.D., Park J., Schulz S., Waldman S.A. (2001). Nucleotide Requirements for CDX2 Binding to the Cis Promoter Element Mediating Intestine-Specific Expression of Guanylyl Cyclase C. FEBS Lett..

[55] Swenson E.S., Mann E.A., Jump M.L., Giannella R.A. (1999). Hepatocyte Nuclear Factor-4 Regulates Intestinal Expression of the guanylin/heat-Stable Toxin Receptor. Am. J. Physiol..

[56] Li P., Lin J.E., Chervoneva I., Schulz S., Waldman S.A., Pitari G.M. (2007). Homeostatic Control of the Crypt-Villus Axis by the Bacterial Enterotoxin Receptor Guanylyl Cyclase C Restricts the Proliferating Compartment in Intestine. Am. J. Pathol..

[57] de Sauvage F.J., Camerato T.R., Goeddel D.V. (1991). Primary Structure and Functional Expression of the Human Receptor for *Escherichia coli* Heat-Stable Enterotoxin. J. Biol. Chem..

[58] Vaandrager A.B. (2002). Structure and Function of the Heat-Stable Enterotoxin receptor/guanylyl Cyclase C. Mol. Cell. Biochem..

[59] Sharma R.K. (2010). Membrane Guanylate Cyclase Is a Beautiful Signal Transduction Machine: Overview. Mol. Cell. Biochem..

[60] van den Akker F., Zhang X., Miyagi M., Huo X., Misono K.S., Yee V.C. (2000). Structure of the Dimerized Hormone-Binding Domain of a Guanylyl-Cyclase-Coupled Receptor. Nature.

[61] Hasegawa M., Matsumoto-Ishikawa Y., Hijikata A., Hidaka Y., Go M., Shimonishi Y. (2005). Disulfide Linkages and a Three-Dimensional Structure Model of the Extracellular Ligand-Binding Domain of Guanylyl Cyclase C. Protein J..

[62] Lauber T., Tidten N., Matecko I., Zeeb M., Rosch P., Marx U.C. (2009). Design and Characterization of a Soluble Fragment of the Extracellular Ligand-Binding Domain of the Peptide Hormone Receptor Guanylyl Cyclase-C. Protein Eng. Des. Sel..

[63] Hasegawa M., Hidaka Y., Matsumoto Y., Sanni T., Shimonishi Y. (1999). Determination of the Binding Site on the Extracellular Domain of Guanylyl Cyclase C to Heat-Stable Enterotoxin. J. Biol. Chem..

[64] Hasegawa M., Shimonishi Y. (2005). Recognition and Signal Transduction Mechanism of *Escherichia coli* Heat-Stable Enterotoxin and Its Receptor, Guanylate Cyclase C. J. Pept. Res..

[65] Biswas K.H., Shenoy A.R., Dutta A., Visweswariah S.S. (2009). The Evolution of Guanylyl Cyclases as Multidomain Proteins: Conserved Features of Kinase-Cyclase Domain Fusions. J. Mol. Evol..

[66] Koller K.J., de Sauvage F.J., Lowe D.G., Goeddel D.V. (1992). Conservation of the Kinaselike Regulatory Domain Is Essential for Activation of the Natriuretic Peptide Receptor Guanylyl Cyclases. Mol. Cell. Biol..

[67] Hanks S.K., Hunter T. (1995). Protein Kinases 6. The Eukaryotic Protein Kinase Superfamily: Kinase (Catalytic) Domain Structure and Classification. FASEB J..

[68] Hirayama T., Wada A., Iwata N., Takasaki S., Shimonishi Y., Takeda Y. (1992). Glycoprotein Receptors for a Heat-Stable Enterotoxin (STh) Produced by Enterotoxigenic *Escherichia coli*. Infect. Immun..

[69] Vaandrager A.B., Schulz S., de Jonge H.R., Garbers D.L. (1993). Guanylyl Cyclase C Is an *N*-Linked Glycoprotein Receptor that Accounts for Multiple Heat-Stable Enterotoxin-Binding Proteins in the Intestine. J. Biol. Chem..

[70] Ghanekar Y., Chandrashaker A., Tatu U., Visweswariah S.S. (2004). Glycosylation of the Receptor Guanylate Cyclase C: Role in Ligand Binding and Catalytic Activity. Biochem. J..

[71] Hasegawa M., Kawano Y., Matsumoto Y., Hidaka Y., Fujii J., Taniguchi N., Wada A., Hirayama T., Shimonishi Y. (1999). Expression and Characterization of the Extracellular Domain of Guanylyl Cyclase C from a Baculovirus and Sf21 Insect Cells. Protein Expr. Purif..

[72] Hasegawa M., Hidaka Y., Wada A., Hirayama T., Shimonishi Y. (1999). The Relevance of *N*-Linked Glycosylation to the Binding of a Ligand to Guanylate Cyclase C. Eur. J. Biochem..

[73] Vaandrager A.B., van der Wiel E., Hom M.L., Luthjens L.H., de Jonge H.R. (1994). Heat-Stable Enterotoxin Receptor/Guanylyl Cyclase C Is an Oligomer Consisting of Functionally Distinct Subunits, which Are Non-Covalently Linked in the Intestine. J. Biol. Chem..

[74] Vijayachandra K., Guruprasad M., Bhandari R., Manjunath U.H., Somesh B.P., Srinivasan N., Suguna K., Visweswariah S.S. (2000). Biochemical Characterization of the Intracellular Domain of the Human Guanylyl Cyclase C Receptor Provides Evidence for a Catalytically Active Homotrimer. Biochemistry.

[75] He X., Chow D., Martick M.M., Garcia K.C. (2001). Allosteric Activation of a Spring-Loaded Natriuretic Peptide Receptor Dimer by Hormone. Science.

[76] Ostedgaard L.S., Baldursson O., Welsh M.J. (2001). Regulation of the Cystic Fibrosis Transmembrane Conductance Regulator Cl^−^ Channel by Its R Domain. J. Biol. Chem..

[77] Vaandrager A.B., Tilly B.C., Smolenski A., Schneider-Rasp S., Bot A.G., Edixhoven M., Scholte B.J., Jarchau T., Walter U., Lohmann S.M., Poller W.C., de Jonge H.R. (1997). CGMP Stimulation of Cystic Fibrosis Transmembrane Conductance Regulator Cl^−^ Channels Co-Expressed with cGMP-Dependent Protein Kinase Type II but Not Type Ibeta. J. Biol. Chem..

[78] Vaandrager A.B., Smolenski A., Tilly B.C., Houtsmuller A.B., Ehlert E.M., Bot A.G., Edixhoven M., Boomaars W.E., Lohmann S.M., de Jonge H.R. (1998). Membrane Targeting of cGMP-Dependent Protein Kinase Is Required for Cystic Fibrosis Transmembrane Conductance Regulator Cl^−^ Channel Activation. Proc. Natl. Acad. Sci. USA.

[79] Chao A.C., de Sauvage F.J., Dong Y.J., Wagner J.A., Goeddel D.V., Gardner P. (1994). Activation of Intestinal CFTR Cl^−^ Channel by Heat-Stable Enterotoxin and Guanylin via cAMP-Dependent Protein Kinase. EMBO J..

[80] Tousson A., Fuller C.M., Benos D.J. (1996). Apical Recruitment of CFTR in T-84 Cells Is Dependent on cAMP and Microtubules but Not Ca^2+^ or Microfilaments. J. Cell Sci..

[81] Kleizen B., Braakman I., de Jonge H.R. (2000). Regulated Trafficking of the CFTR Chloride Channel. Eur. J. Cell Biol..

[82] Golin-Bisello F., Bradbury N., Ameen N. (2005). STa and cGMP Stimulate CFTR Translocation to the Surface of Villus Enterocytes in Rat Jejunum and Is Regulated by Protein Kinase G. Am. J. Physiol. Cell Physiol..

[83] He P., Yun C.C. (2010). Mechanisms of the Regulation of the Intestinal Na^+^/H^+^ Exchanger NHE3. J. Biomed. Biotechnol..

[84] Lucas M.L., Thom M.M., Bradley J.M., O'Reilly N.F., McIlvenny T.J., Nelson Y.B. (2005). *Escherichia coli* Heat Stable (STa) Enterotoxin and the Upper Small Intestine: Lack of Evidence *in Vivo* for Net Fluid Secretion. J. Membr. Biol..

[85] Lucas M.L. (2001). A Reconsideration of the Evidence for *Escherichia coli* STa (Heat Stable) Enterotoxin-Driven Fluid Secretion: A New View of STa Action and a New Paradigm for Fluid Absorption. J. Appl. Microbiol..

[86] Bryant A.P., Busby R.W., Bartolini W.P., Cordero E.A., Hannig G., Kessler M.M., Pierce C.M., Solinga R.M., Tobin J.V., Mahajan-Miklos S. (2010). Linaclotide Is a Potent and Selective Guanylate Cyclase C Agonist that Elicits Pharmacological Effects Locally in the Gastrointestinal Tract. Life Sci..

[87] Eutamene H., Bradesi S., Larauche M., Theodorou V., Beaufrand C., Ohning G., Fioramonti J., Cohen M., Bryant A.P., Kurtz C. (2010). Guanylate Cyclase C-Mediated Antinociceptive Effects of Linaclotide in Rodent Models of Visceral Pain. Neurogastroenterol. Motil..

[88] Sellers Z.M., Childs D., Chow J.Y., Smith A.J., Hogan D.L., Isenberg J.I., Dong H., Barrett K.E., Pratha V.S. (2005). Heat-Stable Enterotoxin of *Escherichia coli* Stimulates a Non-CFTR-Mediated Duodenal Bicarbonate Secretory Pathway. Am. J. Physiol. Gastrointest. Liver Physiol..

[89] Sellers Z.M., Mann E., Smith A., Ko K.H., Giannella R., Cohen M.B., Barrett K.E., Dong H. (2008). Heat-Stable Enterotoxin of *Escherichia coli* (STa) Can Stimulate Duodenal HCO3(-) Secretion via a Novel GC-C- and CFTR-Independent Pathway. FASEB J..

[90] Hugues M., Crane M., Hakki S., O'Hanley P., Waldman S.A. (1991). Identification and Characterization of a New Family of High-Affinity Receptors for *Escherichia coli* Heat-Stable Enterotoxin in Rat Intestinal Membranes. Biochemistry.

[91] Hakki S., Robertson D.C., Waldman S.A. (1993). A 56 kDa Binding Protein for *Escherichia coli* Heat-Stable Enterotoxin Isolated from the Cytoskeleton of Rat Intestinal Membrane Does Not Possess Guanylate Cyclase Activity. Biochim. Biophys. Acta.

[92] Mann E.A., Jump M.L., Wu J., Yee E., Giannella R.A. (1997). Mice Lacking the Guanylyl Cyclase C Receptor Are Resistant to STa-Induced Intestinal Secretion. Biochem. Biophys. Res. Commun..

[93] Schulz S., Lopez M.J., Kuhn M., Garbers D.L. (1997). Disruption of the Guanylyl Cyclase-C Gene Leads to a Paradoxical Phenotype of Viable but Heat-Stable Enterotoxin-Resistant Mice. J. Clin. Invest..

[94] Sindice A., Basoglu C., Cerci A., Hirsch J.R., Potthast R., Kuhn M., Ghanekar Y., Visweswariah S.S., Schlatter E. (2002). Guanylin, Uroguanylin, and Heat-Stable Euterotoxin Activate Guanylate Cyclase C and/or a Pertussis Toxin-Sensitive G Protein in Human Proximal Tubule Cells. J. Biol. Chem..

[95] Carrithers S.L., Ott C.E., Hill M.J., Johnson B.R., Cai W., Chang J.J., Shah R.G., Sun C., Mann E.A., Fonteles M.C. (2004). Guanylin and Uroguanylin Induce Natriuresis in Mice Lacking Guanylyl Cyclase-C Receptor. Kidney Int..

[96] Steinbrecher K.A., Tuohy T.M., Goss K.H., Scott M.C., Witte D.P., Groden J., Cohen M.B. (2000). Expression of Guanylin Is Downregulated in Mouse and Human Intestinal Adenomas. Biochem. Biophys. Res. Commun..

[97] Carrithers S.L., Parkinson S.J., Goldstein S., Park P., Robertson D.C., Waldman S.A. (1994). *Escherichia coli* Heat-Stable Toxin Receptors in Human Colonic Tumors. Gastroenterology.

[98] Carrithers S.L., Barber M.T., Biswas S., Parkinson S.J., Park P.K., Goldstein S.D., Waldman S.A. (1996). Guanylyl Cyclase C Is a Selective Marker for Metastatic Colorectal Tumors in Human Extraintestinal Tissues. Proc. Natl. Acad. Sci. USA.

[99] Schulz S., Hyslop T., Haaf J., Bonaccorso C., Nielsen K., Witek M.E., Birbe R., Palazzo J., Weinberg D., Waldman S.A. (2006). A Validated Quantitative Assay to Detect Occult Micrometastases by Reverse Transcriptase-Polymerase Chain Reaction of Guanylyl Cyclase C in Patients with Colorectal Cancer. Clin. Cancer Res..

[100] Waldman S.A., Hyslop T., Schulz S., Barkun A., Nielsen K., Haaf J., Bonaccorso C., Li Y., Weinberg D.S. (2009). Association of GUCY2C Expression in Lymph Nodes with Time to Recurrence and Disease-Free Survival in pN0 Colorectal Cancer. JAMA.

[101] Snook A.E., Stafford B.J., Li P., Tan G., Huang L., Birbe R., Schulz S., Schnell M.J., Thakur M., Rothstein J.L. (2008). Guanylyl Cyclase C-Induced Immunotherapeutic Responses Opposing Tumor Metastases without Autoimmunity. J. Natl. Cancer Inst..

[102] Wolfe H.R., Mendizabal M., Lleong E., Cuthbertson A., Desai V., Pullan S., Fujii D.K., Morrison M., Pither R., Waldman S.A. (2002). *In Vivo* Imaging of Human Colon Cancer Xenografts in Immunodeficient Mice using a Guanylyl Cyclase C—Specific Ligand. J. Nucl. Med..

[103] Giblin M.F., Gali H., Sieckman G.L., Owen N.K., Hoffman T.J., Forte L.R., Volkert W.A. (2004). *In Vitro* and *in Vivo* Comparison of Human *Escherichia coli* Heat-Stable Peptide Analogues Incorporating the 111In-DOTA Group and Distinct Linker Moieties. Bioconjug. Chem..

[104] Giblin M.F., Sieckman G.L., Watkinson L.D., Daibes-Figueroa S., Hoffman T.J., Forte L.R., Volkert W.A. (2006). Selective Targeting of *E. coli* Heat-Stable Enterotoxin Analogs to Human Colon Cancer Cells. Anticancer Res..

[105] Giblin M.F., Sieckman G.L., Shelton T.D., Hoffman T.J., Forte L.R., Volkert W.A. (2006). *In Vitro* and *in Vivo* Evaluation of 177Lu- and 90Y-Labeled *E. coli* Heat-Stable Enterotoxin for Specific Targeting of Uroguanylin Receptors on Human Colon Cancers. Nucl. Med. Biol..

[106] Giblin M.F., Gali H., Sieckman G.L., Owen N.K., Hoffman T.J., Volkert W.A., Forte L.R. (2006). *In Vitro* and *in Vivo* Evaluation of 111In-Labeled *E. coli* Heat-Stable Enterotoxin Analogs for Specific Targeting of Human Breast Cancers. Breast Cancer Res. Treat..

[107] Tian X., Michal A.M., Li P., Wolfe H.R., Waldman S.A., Wickstrom E. (2008). STa Peptide Analogs for Probing Guanylyl Cyclase C. Biopolymers.

[108] Liu D., Overbey D., Watkinson L.D., Daibes-Figueroa S., Hoffman T.J., Forte L.R., Volkert W.A., Giblin M.F. (2009). *In Vivo* Imaging of Human Colorectal Cancer using Radiolabeled Analogs of the Uroguanylin Peptide Hormone. Anticancer Res..

[109] Urbanski R., Carrithers S.L., Waldman S.A. (1995). Internalization of *E. coli* ST Mediated by Guanylyl Cyclase C in T84 Human Colon Carcinoma Cells. Biochim. Biophys. Acta.

[110] Pitari G.M., Zingman L.V., Hodgson D.M., Alekseev A.E., Kazerounian S., Bienengraeber M., Hajnoczky G., Terzic A., Waldman S.A. (2003). Bacterial Enterotoxins Are Associated with Resistance to Colon Cancer. Proc. Natl. Acad. Sci. USA.

[111] Parkin D.M., Bray F., Ferlay J., Pisani P. (2005). Global Cancer Statistics, 2002. CA Cancer J. Clin..

[112] Pitari G.M., Di Guglielmo M.D., Park J., Schulz S., Waldman S.A. (2001). Guanylyl Cyclase C Agonists Regulate Progression through the Cell Cycle of Human Colon Carcinoma Cells. Proc. Natl. Acad. Sci. USA.

[113] Pitari G.M., Lin J.E., Shah F.J., Lubbe W.J., Zuzga D.S., Li P., Schulz S., Waldman S.A. (2008). Enterotoxin Preconditioning Restores Calcium-Sensing Receptor-Mediated Cytostasis in Colon Cancer Cells. Carcinogenesis.

[114] Li P., Lin J.E., Snook A.E., Gibbons A.V., Zuzga D.S., Schulz S., Pitari G.M., Waldman S.A. (2008). Colorectal Cancer Is a Paracrine Deficiency Syndrome Amenable to Oral Hormone Replacement Therapy. Clin. Transl. Sci..

[115] Birbe R., Palazzo J.P., Walters R., Weinberg D., Schulz S., Waldman S.A. (2005). Guanylyl Cyclase C Is a Marker of Intestinal Metaplasia, Dysplasia, and Adenocarcinoma of the Gastrointestinal Tract. Hum. Pathol..

[116] Witek M.E., Nielsen K., Walters R., Hyslop T., Palazzo J., Schulz S., Waldman S.A. (2005). The Putative Tumor Suppressor Cdx2 Is Overexpressed by Human Colorectal Adenocarcinomas. Clin. Cancer Res..

[117] Li P., Waldman S.A. (2010). Corruption of Homeostatic Mechanisms in the Guanylyl Cyclase C Signaling Pathway Underlying Colorectal Tumorigenesis. Cancer Biol. Ther..

[118] Lin J.E., Li P., Snook A.E., Schulz S., Dasgupta A., Hyslop T.M., Gibbons A.V., Marszlowicz G., Pitari G.M., Waldman S.A. (2010). The Hormone Receptor GUCY2C Suppresses Intestinal Tumor Formation by Inhibiting AKT Signaling. Gastroenterology.

[119] Li P., Lin J.E., Marszlowicz G.P., Valentino M.A., Chang C., Schulz S., Pitari G.M., Waldman S.A. (2009). GCC Signaling in Colorectal Cancer: Is Colorectal Cancer a Paracrine Deficiency Syndrome?. Drug News Perspect..

[120] Shailubhai K., Yu H.H., Karunanandaa K., Wang J.Y., Eber S.L., Wang Y., Joo N.S., Kim H.D., Miedema B.W., Abbas S.Z., Boddupalli S.S., Currie M.G., Forte L.R. (2000). Uroguanylin Treatment Suppresses Polyp Formation in the Apc(Min/+) Mouse and Induces Apoptosis in Human Colon Adenocarcinoma Cells via Cyclic GMP. Cancer Res..

[121] Pitari G.M., Baksh R.I., Harris D.M., Li P., Kazerounian S., Waldman S.A. (2005). Interruption of Homologous Desensitization in Cyclic Guanosine 3',5'-Monophosphate Signaling Restores Colon Cancer Cytostasis by Bacterial Enterotoxins. Cancer Res..

